# Radiation-Induced Secondary Cancer Risk Assessment in Patients With Lung Cancer After Stereotactic Body Radiotherapy Using the CyberKnife M6 System With Lung-Optimized Treatment

**DOI:** 10.3389/fbioe.2020.00306

**Published:** 2020-05-07

**Authors:** Pei-Ju Chao, I-Hsing Tsai, Chun-Chieh Huang, Chih-Hsueh Lin, Chin-Shiuh Shieh, Yang-Wei Hsieh, Pei-Ying Yang, Hsiao-Fei Lee, Tsair-Fwu Lee

**Affiliations:** ^1^Medical Physics and Informatics Laboratory of Electronics Engineering, National Kaohsiung University of Science and Technology, Kaohsiung, Taiwan; ^2^Department of Radiation Oncology, Kaohsiung Chang Gung Memorial Hospital and Chang Gung University College of Medicine, Kaohsiung, Taiwan; ^3^Biomedical Engineering, Kaohsiung Medical University, Kaohsiung, Taiwan

**Keywords:** lung cancer, SBRT, SCR, OED, EAR, LAR

## Abstract

**Background:**

To evaluate the lifetime secondary cancer risk (SCR) of stereotactic body radiotherapy (SBRT) using the CyberKnife (CK) M6 system with a lung-optimized treatment (LOT) module for lung cancer patients.

**Methods:**

We retrospectively enrolled 11 lung cancer patients curatively treated with SBRT using the CK M6 robotic radiosurgery system. The planning treatment volume (PTV) and common organs at risk (OARs) for SCR analysis included the spinal cord, total lung, and healthy normal lung tissue (total lung volume - PTV). Schneider’s full model was used to calculate SCR according to the concept of organ equivalent dose (OED).

**Results:**

CK-LOT-SBRT delivers precisely targeted radiation doses to lung cancers and achieves good PTV coverage and conformal dose distribution, thus posing limited SCR to surrounding tissues. The three OARs had similar risk equivalent dose (RED) values among four different models. However, for the PTV, differences in RED values were observed among the models. The cumulative excess absolute risk (EAR) value for the normal lung, spinal cord, and PTV was 70.47 (per 10,000 person-years). Schneider’s Lnt model seemed to overestimate the EAR/lifetime attributable risk (LAR).

**Conclusion:**

For lung cancer patients treated with CK-LOT optimized with the Monte Carlo algorithm, the SCR might be lower. Younger patients had a greater SCR, although the dose–response relationship seemed be non-linear for the investigated organs, especially with respect to the PTV. Despite the etiological association, the SCR after CK-LOT-SBRT for carcinoma and sarcoma, is low, but not equal to zero. Further research is required to understand and to show the lung SBRT SCR comparisons and differences across different modalities with motion management strategies.

## Introduction

Stereotactic body radiotherapy (SBRT) is an external beam radiotherapy method for accurately delivering high-dose (∼6–30 Gy), highly localized, and conformal radiation in five or fewer fractions with steep dose gradients (Benedict et al., 2010; Guckenberger et al., 2014; Ceniceros et al., 2016). This is an attractive, non-invasive, well-tolerated outpatient treatment option for lung cancers (Guckenberger et al., 2019). Treatment of lung cancer patients with SBRT has the benefit of excellent local control rates (Nagata et al., 2005; Onishi et al., 2007). In our department, lung cancers are treated using a CyberKnife (CK) M6 robotic radiosurgery system with a lung-optimized treatment (LOT) module (Accuray Inc., Sunnyvale, CA, United States), because it has unique characteristics that make it suitable for SBRT of lung tumors (Brown et al., 2008; Jang et al., 2016).

Secondary cancer risk (SCR) in patients with lung cancer cannot be ignored and is highly correlated with radiation treatment. Patients treated with SBRT are at risk of developing secondary cancer during their lifetime, which may directly affect treatment decisions and quality of life (Lan et al., 2019). However, unless an atomic bomb explosion were to occur, it is not possible to carry out human experiments for SCR studies. Of note, the timing of the development of secondary neoplasm depends on histology; specifically, the latency period for benign tumors is approximately 15–20 years following radiotherapy, whereas it is approximately 9–14 years in the case of malignant tumors (Ron et al., 1988; Patel and Chiang, 2014). Due to time limitations and restricted clinical availability, and as such studies require decades rather than years to finish, SCR modeling is becoming popular in this field.

The original BEIR VII models for SCR analysis are based on epidemiological statistics of atomic bomb explosions, which constitute one-shot low-dose radiation exposure (Council, 2006). Therefore Schneider and Walsh (2008), Schneider et al. (2011a,b) took into consideration the distribution of non-uniform dose and high-dose regions to propose a more suitable model for radiotherapy. They fitted data based on Hodgkin’s disease to develop a full model that takes into account the balance between cell killing and repopulation (Schneider and Walsh, 2008; Schneider et al., 2011a, b). Moreover, Schneider et al. proposed the concept of OED, which was used to model the specific phenomenological correlates of SCR, including cell killing, repopulation, and proliferation. Then, the data were fitted into linear, linear-exponential, plateau, and full models for the SCR analysis of the dose–response effect (Schneider and Walsh, 2008; Schneider et al., 2011a, b). However, the original model is based on fractional radiotherapy. In this study, for the case of SBRT, a hypofraction RT is explored, and it is necessary to use BED to translate the results, to measure the same radiation biological effect so that we can reasonably assess the incidence of CK-LOT-SBRT-induced SCR. Schneider’s SCR models took into account the interaction between cell death and repopulation (Schneider et al., 2011a), so they could be used to investigate the risk of secondary cancer in CK-LOT-SBRT. All corresponding dose–volume histograms (DVHs) extracted from the planning system were adjusted to account for non-homogeneous organ dose distributions in higher dose areas.

To our knowledge, there have been few reports analyzing SCR in stereotactic body lung radiotherapy patients treated using a CK M6 robotic radiosurgery system. Thus, our goal was to evaluate radiation-induced SCR in patients with lung tumors after CK-LOT-SBRT; dose–response modeling took account of the concepts of organ equivalent dose (OED), excess absolute risk (EAR), and lifetime attributable risk (LAR).

## Materials and Methods

### Patients

During the period of the study, 11 lung cancer patients curatively treated by SBRT using the CK M6 robotic radiosurgery system equipped with LOT module (CK-LOT-SBRT), together with the real-time image-guided Synchrony Respiratory Tracking System (Accuray, Inc.) to deal with target movement, were enrolled retrospectively. An IRIS collimator (variable aperture collimator; Accuray, Inc.) optimized by the Monte Carlo (MC) algorithm was used in all cases. The characteristics of the lesions and associated dosimetric values are listed in Table 1. All patients had a median planning treatment volume (PTV) of 73.37 cm^3^ (range: 6.69–203.71 cm^3^). To assess the age dependence of SCR, patients of various ages were enrolled; the mean age was 58 years (range: 38–68 years). All patients were of clinical stage I–II, according to the American Joint Committee on Cancer (AJCC 8^*th*^ edition) staging system.

**TABLE 1:** Characteristics of lesions and associated dosimetric values.

Patients	Exposure age (years)	Gender	PTV (cm^3^)	Normal lung (cm^3^)	CI	nCI	HI	Coverage (%)	Prescribed Dose (Gy)
1	38	M	12.64	2555.96	2.17	2.24	1.35	96.89	40
2	48	F	186.00	1523.04	1.34	1.40	1.33	95.93	40
3	53	M	69.53	4560.55	1.54	1.60	1.37	96.37	40
4	57	F	6.69	2743.08	1.48	1.51	1.33	97.93	55
5	59	M	46.85	2535.69	1.39	1.47	1.23	94.70	40
6	60	M	203.71	4025.91	1.30	1.36	1.28	95.81	40
7	62	M	45.33	4254.25	1.26	1.32	1.39	95.29	50
8	64	M	9.98	3804.36	1.22	1.27	1.19	96.31	50
9	65	M	73.99	3143.92	1.49	1.55	1.33	96.28	40
10	66	F	97.29	2664.68	1.40	1.46	1.28	95.75	40
11	68	M	55.08	3300.08	1.37	1.42	1.23	96.44	40
Mean	58 ± 8.50	—	73.37 ± 63.42	3191.96 ± 862.66	1.45 ± 0.25	1.51 ± 0.25	1.30 ± 0.06	96.15 ± 0.80	43.18 ± 5.60

*CI, conformal index; nCI, new conformal index; HI: homogeneity index; PTV, planning target volume.*

### Ethical Approval and Informed Consent

The institutional review board of Chang Gung Memorial Hospital approved this study (IRB approval No. 201802377B0), and the requirement for informed consent was waived given the retrospective nature of the study.

### Treatment Planning

Patients were immobilized with a vacuum bag while wearing a Synchrony Vest (Accuracy, Inc.). Two series of thoracic computed tomography (CT) images (0.9375 × 0.9375 × 1.25 mm^3^; 512 × 512 pixels per slice; slice thickness, 1.25 mm) for LOT planning were acquired (full inhalation and exhalation breath-hold images) using a LightSpeed RT16 instrument (GE Medical Systems, Milwaukee, WI, United States).

MultiPlan Treatment Planning Software (MTPS; version 5.1.3; Accuray Inc.) was used to generate the treatment plans in conjunction with the LOT module and real-time image-guided Synchrony Respiratory Tracking System; tissue density heterogeneity in the lung was corrected for. The gross tumor volume (GTV) and organs at risk (OARs) were identified and analyzed via lung and mediastinum windows by the same radiation oncologist. The GTV margins were expanded by 2–3 mm in the tracked direction, and by 5–8 mm in the untracked direction, to establish the PTV. The OARs were contoured and defined according to the RTOG 0236 protocol (Xiao et al., 2009), which included the lungs, spinal cord, heart, and esophagus. The plans were optimized by applying the MC algorithm for dose calculation, which enhances the accuracy of the dose calculation when modeling the interactions among individual photons, such that accurate dose distributions can be achieved when simulating many types of events (Gibbs and Loo Jr., 2010). The LOT module of the Cyberknife Xsight Lung Tracking System (Accuracy, Inc.) allows for the application of fiducial-free motion management strategies (Ricotti et al., 2018). During CK treatment delivery, the Synchrony Respiratory Tracking System allows for real-time tracking of the target, which changes position due to movement occurring during breathing (Collins et al., 2009, 2012). Motion correlations of the internal tumor locations on two images, acquired with two in-room orthogonal X-rays, can be measured, with the external respiratory signal arising from three light-emitting diodes (LEDs) being fixed on the patient thorax. The details can be found in previous studies (Collins et al., 2007, 2009, 2012).

The radiation dose was prescribed to ∼80% of the isodose line of the PTV, covering ≥ 95% of the volume. The maximum dose was defined by the 100% isodose line. The prescribed dose was 40–55 Gy, delivered in five fractions, covering the GTV and regions exhibiting microscopic disease. The biologically effective dose (BED) conversion factor applied to the PTV was α/β = 10 Gy/fraction; for the OARs, it was α/β = 3 Gy for 2 Gy/fraction.

The conformity index (CI), new conformity index (nCI), homogeneity index (HI), and PTV coverage values were obtained from the MTPS. In the CK M6 system, a pair of orthogonal kV X-ray imaging systems were used for simultaneous target tracing (Jang et al., 2016; Lan et al., 2019).

### SCR Assessment

The PTV and OARs commonly used for SCR analysis include the spinal cord, total lung, and healthy normal lung tissue (total lung volume - PTV) because radiation-induced secondary cancers usually occur in the beam-bordering region (Harbron et al., 2014). To calculate SCRs, DVHs were extracted from the MTPS. To measure non-uniform organ dose distribution in high-dose areas, the OED concept was applied. Secondary carcinomas and sarcomas were modeled separately; previous studies provide detailed information (Schneider and Kaser-Hotz, 2005; Zwahlen et al., 2009; Abo-Madyan et al., 2014; Lee et al., 2018), and a brief description is presented below.

The concept of OED, first reported by Schneider et al., was used to model the specific phenomenological correlates of SCR (Schneider and Kaser-Hotz, 2005), including repopulation, proliferation, and cell killing. Data are fitted to the OED using linear, linear-exponential, plateau, and full models of dose–response.

The OED is used as a proxy for the risk of radiation-induced carcinoma within the OARs (Equation 1). The PTV region was analyzed using a sarcoma induction model (Equation 2). Based on the total dose (D), cell killing (α), dose per fraction (dF), and repopulation (R) parameters, the OED for carcinoma induction can be derived as follows (Schneider et al., 2011a):

(1) $$OED_{c}=\frac{1}{V}\sum_{i}V_{i}\cdot \frac{e^{-\alpha_{i}^{\prime }\,D_{i}}}{\alpha_{i}^{\prime }\,R}(1-2R+R^{2}e^{\alpha_{i}^{\prime }\,D_{i}}-{\left(1-R\right)}^{2}-e^{\frac{\alpha_{i}^{\prime }\,R}{1-R}\,D_{i}}$$

The OED for the sarcoma induction model is derived as follows:

(2) $$OED_{s}=\frac{1}{V}\sum_{i}V_{i}\cdot \frac{e^{-\alpha_{i}^{\prime }\,D_{i}}}{\alpha_{i}^{\prime }\,R}(1-2R+R^{2}e^{\alpha_{i}^{\prime }\,D_{i}}-\alpha_{i}^{\prime }RD_{i}-{\left(1-R\right)}^{2}-e^{\frac{\alpha_{i}^{\prime }\,R}{1-R}\,D_{i}})$$

(3) $$\alpha^{\prime }=\alpha +\beta \,D_{i}\cdot \frac{d_{F}}{D}$$

We calculated the risk equivalent dose (RED) in the organs, which refers to the dose–response relationship of the point dose rather than the organ dose, where *i* is the bin number of the DVH. The OED can be determined by dividing the sum of the RED values of all voxels by the number of voxels (*N*), where *V* is the total organ volume. Table 2 lists the data for all model parameters in the EAR and Schneider OED models. The per 10,000 PY values of γ_*e*_, γ_*a*_, β, and *R* were obtained based on previous studies (Schneider et al., 2011a, b).

**TABLE 2:** Summary of risk parameters used for the corresponding dose-response models.

Carcinoma												

Organ\model	Lnt	LinExp			Plateau			Full model			Age parameters	
					
	β	β	α	*R*	β	α	*R*	β	α	*R*	γ_*e*_	γ_*a*_
Spinal cord	0.44	0.51	0.009	0	0.51	0.021	1	0.51	0.018	0.93	−0.024	2.38
Lung	7.5	7.5	0.022	0	7.5	0.056	1	7.5	0.042	0.83	0.002	4.23

**Sarcoma**												

**Organ\model**	**Lnt**	**Low repopulation**			**Intermediate repopulation**			**Full tissue recovery**			**Age parameters**	
					
	**β**	**β**	**α**	***R***	**β**	**α**	***R***	**β**	**α**	***R***	**γ_*e*_**	**γ_*a*_**

PTV	0.39	3.3	0.040	0.1	0.6	0.060	0.5	0.35	0.093	1	−0.013	−0.56

*β is used for EAR calculation only; β within the α/β ratio is calculated from a based on α/β = 3 Gy for all tissues; R, cell repopulation parameter; α, cell killed parameter; Plateau, Plateau dose response model; Full model, Schneider’s parameterization (full) model; γe and γa, modifying factors for age; Lnt, Linear-no-threshold dose response model; LinExp, linear-exponential dose response model.*

There is currently much debate about the model’s function shape of the dose–response curve for radiation-induced cancer. It is not known whether the cancer risk as a function of dose remains linear due to cell killing, or decreases at high doses, or stabilizes due to, for example, the balance between reproductive effects and cell killing. The repopulation/repair parameter, *R*, characterizes the repopulation/repair capability of the tissue between two dose fractions, which is 1 if complete repopulation/repair occurs, and is equal to 0 otherwise. Among different cell types, the risk of sarcoma overdose observed in the study of atomic bomb survivors is an order of magnitude smaller than that of carcinoma. However, data from radiation-treated patients indicate that high-dose sarcoma induction is similar to carcinoma induction. Therefore, it is inappropriate to assume a pure linear dose–response relationship for the induction of sarcoma. No model is exactly correct, so we used different models for reference.

The EAR is defined as the product of the OED and the initial slope of the dose–response curve in the low-dose region (excess cancers per 10,000 PY) (Equation 4). Preston et al. introduced a parametric method for determining the initial slope using data from atomic bomb survivors (Preston et al., 2007).

(4) E⁢A⁢R⁢(D,e,a,s)=O⁢E⁢D⋅β⋅e(γ[e-30]e+γlan[a70])⁢(1±s)

The model parameters γ_*e*_, γ_*a*_, and β for a given organ (excess cases per 10,000 PY) were obtained from Schneider et al. (2011a) and are presented in Table 2. γ_*e*_ and γ_*a*_ are age correction factors, and β denotes the initial slope of the dose–response model in the low-dose region (note that this β is not the same as that in the cell survival curve in Equation 3). The parameters *e* and *a* represent the age of exposure and attained age, respectively; for parameter *e*, we considered the attained age in the 20 years after treatment (Paganetti et al., 2012). *S* is gender, where female is +0.17 and male is −0.17. Equation 5 was used to calculate LAR and denotes the lifetime likelihood (%) of a second cancer (expressed as a multiple of baseline risk). For SCR analysis, LAR is an effective measurement because it considers the age and predicted lifespan of the patient at the time of exposure (Moteabbed et al., 2014).

(5) $$L\,A\,R\,\left(D,e,a\right)=\int_{a=e+L}^{75}E\,A\,R\,\left(D,e,a,s\right)\cdot \frac{S\,\left(a\right)}{S\,\left(e\right)}\,da$$

The EAR is calculated based on the incubation period of solid cancer and the attained age after treatment. L is the incubation period of solid cancer and is set to 5–70 years (Council, 2006). *S*(*a*) represents the surviving population at the time of treatment and *S*(*e*) represents the attained age after treatment (Kellerer et al., 2001). The survival probability used in this study is derived from the life table of a Taiwanese population (Ministry of the Interior, 2014).

### BEIR VII Model

The BEIR VII model was used to evaluate the SCR with respect to sarcoma and carcinoma. Details can be found in previous studies (Schneider et al., 2011a; Lan et al., 2019). In this model, a linear dose function is used to derive the cancer-induced dose–response relationship.

**FIGURE 1:**
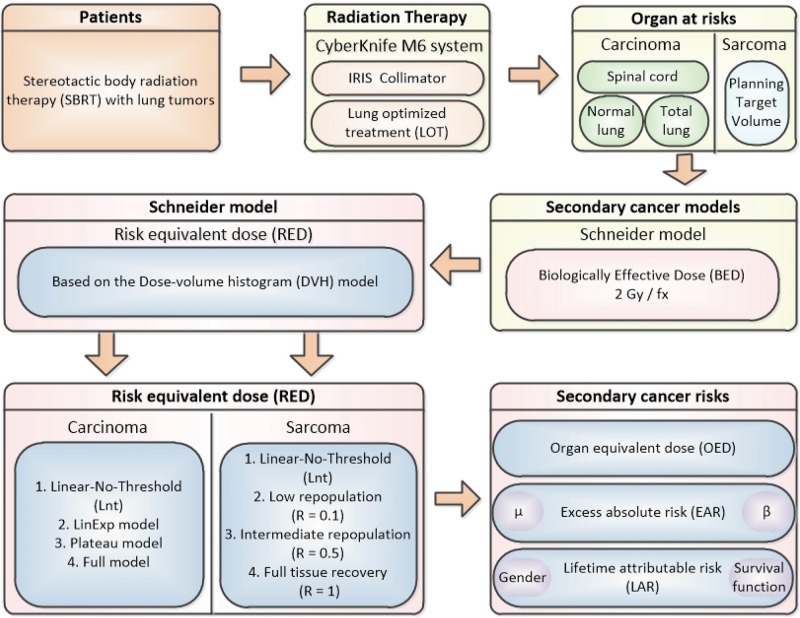
Flow chart. EAR, excess absolute risk.

Figure 1 shows the flow chart for this study process.

## Results

**FIGURE 2:**
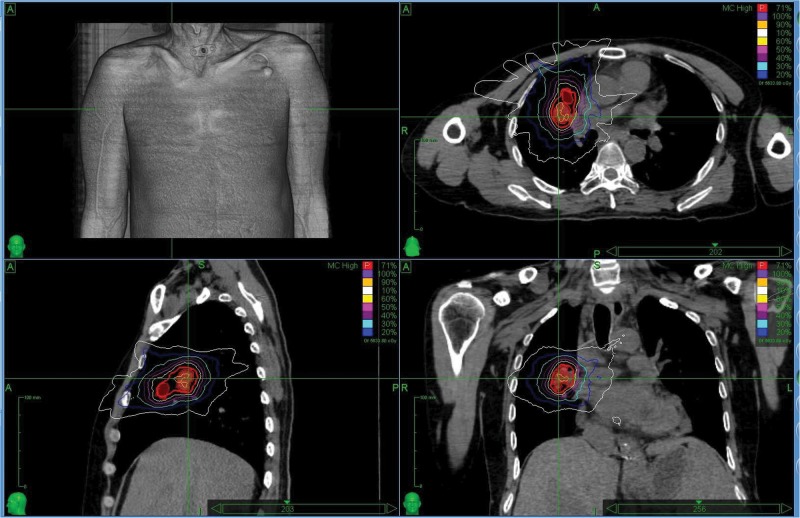
The isodose distributions on transverse, sagittal, and coronal views for one representative sample. MLC, Multileaf collimator; IRIS, Iris collimator.

The characteristics of the patients and associated dosimetric values are listed in Table 1. The patients are numbered according to exposure age. The tumor volume ranged from 6.69 to 203.71 cm^3^ (median = 55.08). The dosimetric value for the PTV coverage in the CK-LOT-SBRT plans was 96.15 ± 0.80, and the CI was 1.45 ± 0.25. The nCI was 1.51 ± 0.25, and the HI was 1.30 ± 0.06 (Table 1). Figure 2 presents the isodose distributions in the transverse, coronal, and sagittal views for a single representative sample.

**FIGURE 3:**
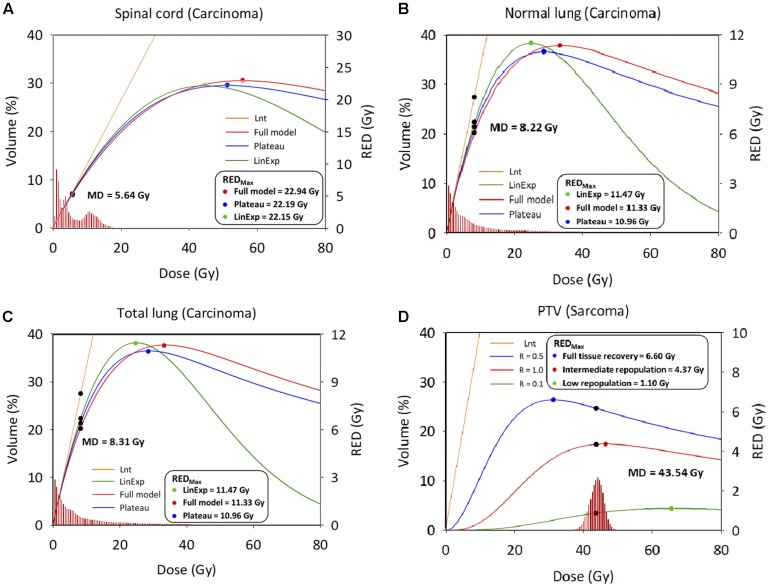
Differential dose volume histograms and risk equivalent dose graph. **(A)** Spinal cord. **(B)** Normal lung. **(C)** Total lung; *X* axis: Dose; *Y* axis: Volume (Left), risk-equivalent dose (Right); Unit is percentage (Left) and Gy (Right). DVH, dose–volume histogram; MD, mean dose, Gy; PTV, planning target volume; R, repopulation parameter; RED, risk-equivalent dose, Gy; RED_*max*_, maximum point of the RED curve; Lnt, linear-no-threshold dose response model; LinExp, linear-exponential dose–response model; Plateau, plateau dose–response model; Full, Schneider parameterization dose–response model; low repopulation (L*_*R*_*_=__0.1_) R = 0.1, intermediate repopulation (I*_*R*_*_=__0.5_) *R* = 0.5, full tissue recovery models (*F*_*R*__=__1.0_) *R* = 1.0.

Figures 3A–D show the relationship between the differential DVH plots and RED values. The three OARs had similar RED values among the four different models, as reflected in the RED curves (left side of the maximum dose–response RED curve [RED_*max*_]), which would indicate a lower SCR. We observed differences in RED values for PTV due to the large low-dose volumes exposed. The SCR depends on the association between the volume exposed in the differential DVHs and the dose–response curves.

**FIGURE 4:**
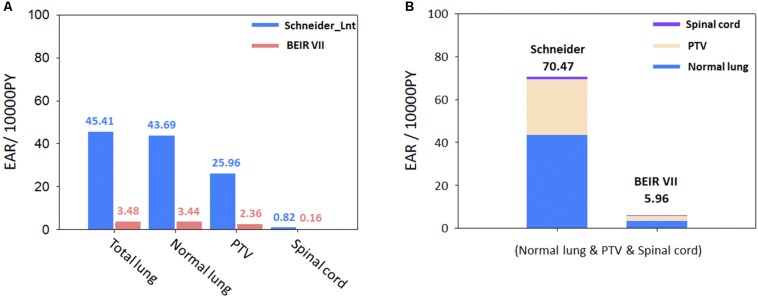
**(A)** Schneider Lnt model EAR for selected organs. **(B)** Schneider Lnt and BEIR VII cumulative EAR of cancer for the three OARs at age 75 years (normal lung + spinal cord + PTV); BED: BED conversion; unit is per 10,000 person-years (PY). EAR, excess absolute risk; Lnt, linear-no-threshold model; PTV, planning target volume.

The cumulative estimated values of EAR for the 11 patients are presented in Figures 4A,B, using Schneider’s Lnt model and the BEIR VII model, to determine the SCR for the OARs. The cumulative EAR for the normal lung, spinal cord, and PTV was 70.47 (per 10,000 PY). The EAR for the total lung was ∼1.75 times higher than that for the PTV. The results indicate that SCR may be higher if calculated using Schneider’s Lnt model; in this study, the SCR was about 11-fold higher using Schneider’s model relative to the BEIR VII model.

**FIGURE 5:**
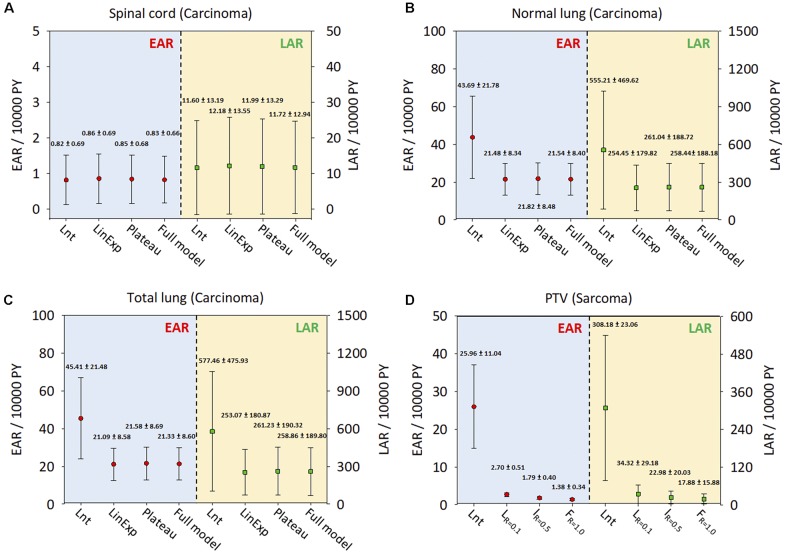
The EARs and LARs of 11 patients (mean and standard deviation) for the investigated organs. **(A)** Spinal cord. **(B)** Normal lung. **(C)** Total lung. **(D)** PTV. The EAR and LAR have units of excess cases per 10,000 person-years (PY). Lnt, linear-no-threshold dose–response model; LinExp, linear-exponential dose–response model; Plateau, plateau dose–response model; Full, Schneider parameterization dose–response model; Low repopulation (L*_*R*_*_=__0.1_) *R* = 0.1, Intermediate repopulation (I*_*R*_*_=__0.5_) *R* = 0.5, Full tissue recovery models (F*_*R*_*_=__1.0_) *R* = 1.0; EAR, excess absolute risk; LAR, lifetime attributable risk; Lnt, linear-no-threshold model; PTV, planning target volume.

Figures 5A–C show the ranges of EAR/LAR values derived using the Lnt, linear-exponential, plateau, and full models of carcinoma induction in the spinal cord, normal lung, and total lung. Figure 5D shows the risk of sarcoma induction in the PTV using Schneider’s Lnt model, which seemed to overestimate the EAR/LAR. Supplementary Figures S1–S4 present the LAR and OED data for each dose–response model. The results showed that the LAR depends on the patient’s characteristics. Although the patients had an almost identical OED, albeit with different LARs, younger patients had a greater SCR. The dose–response relationship seems be non-linear for the investigated organs, especially in the case of the PTV.

## Discussion

The IRIS collimator was used for all patients treated using CK-LOT-SBRT, with the MC algorithm applied. The main reason for not applying the InCise multileaf collimator (MLC) is that the MC algorithm is not included in MLC mode. CK-LOT-SBRT delivers precisely targeted radiation doses to lung cancers and achieves good PTV coverage and homogeneous dose distribution, with a low SCR for surrounding tissues. The dosimetric values for the CK-LOT-SBRT plans, for CI, nCI, and HI, were similar to those used in previous studies by Swangsilpa et al. (2012) and Lischalk et al. (2016); a smaller value is considered beneficial (Table 1). A similar report by Brown et al. demonstrated that it is feasible and safe to deliver precisely targeted hypofractionated radiation to lung tumors (Brown et al., 2008).

This study assessed the SCR for carcinoma using all available models, i.e., linear, plateau, linear-exponential, and full models, where there is still a lack of understanding of the dose–response relationship for patients exposed to CK-LOT-SBRT. The OED values were almost identical after considering the contribution of irradiation, except for the Lnt model, because the OED values were small and the models were all concerned with the linear low-dose region. Regarding the SCR for sarcoma, the PTV showed a large difference between the Lnt model and the other three models. Repopulation, proliferation, and cell killing should be considered to ensure accurate evaluation of SCR. We overlaid the RED values on the DVH curve (differential) of a representative patient to determine the correlation between the DVH and RED values (Figure 3). The RED_*max*_ was plotted; the left side of the RED_*max*_ curve is steeper than that the right side. The mean doses for the three investigated organs all lay on the left side of the RED_*max*_ curve, which indicates sensitivity to a small dose increase. SCR varies with the dose–volume distribution (Lee et al., 2018).

Since the lung has a higher beta (β) value than all other organs, it is sensitive to radiation even at low doses (Lan et al., 2019). In the present study, the Schneider Lnt model had a larger cumulative EAR value than the BEIR VII model (see Figure 4B). The BEIR VII model might underestimate SCR because it does not consider high-dose radiation-induced cancer, or the distribution of non-uniform doses; also, it does not correct for the BED. In our opinion, Schneider’s models with BED correction can be used to determine the incidence of CK-LOT-SBRT-induced SCR. Preston et al. (2007) demonstrated that the lungs are more likely to be affected by radiation-induced cancers than other organs. Particular attention should be paid to carcinomas near the treatment area, given their potential for secondary malignancy (Lan et al., 2019). Hall (2004) and Xu et al. (2008) reported a dose–response relationship for radiation-induced carcinogenesis in humans. The ideal does range is ∼0.1–2.5 Gy. In high-dose radiation therapy, uncertainty exists regarding the dose–response relationship. Our study showed similar results to Xu et al. (2008) for the spinal cord, normal lung, and total lung with respect to the RED values, which were in the linear region because the patients received low-to-intermediate doses. However, the PTV received a higher dose than other regions, such that the RED differed among the various models. As there is no recognized optimal dose range for the PTV, the selection of parameters or models for SCR analysis should consider the uncertainty of the dose–response relationship.

The exposure dose and age at initial treatment may affect the likelihood of radiation-induced secondary cancers, as shown by Wei et al. (2012). Patients with early stage lung cancer undergoing SBRT or lobectomy have a good chance of a curative outcome. SCR is a long-term non-negligible side effect in young patients, especially long-term survivors of lung cancer. The current results showed that younger patients had higher SCR, but the dose–response relationship appeared to be non-linear for all organs investigated, especially in the case of the PTV (Supplementary Figures S1–S4).

Overall, SCR data for CK-LOT-SBRT are lacking, and it may take decades to address this gap (Lan et al., 2019), to confirm new treatment technologies, not only to provide good PTV coverage but also to reduce the SCR risk. Steneker et al. (2006) conducted a comparative treatment-planning study between intensity-modulated photon and proton therapy to investigate the risks of secondary cancer induction on five head and neck patients. The parameters of interest were also fitted into a Schneider model, a system based on atomic bomb and Hodgkin’s disease. They found that in five patients with squamous cell carcinoma increased, the average risk of a second solid tumor increased by about 1.5 times when compared with intensity-modulated proton therapy (Steneker et al., 2006). Even if the patient number is small, it also indicates the prognosis status of the new technology. Moreover, Moteabbed et al. (2014) compared six pediatric patients with brain tumors in different treatment modalities. Abo-Madyan et al. (2014) scanned 10 representative breast cancer patients using different treatment techniques. Kim et al. (2014) reported the risk of secondary cancers from scattered radiation during intensity-modulated radiotherapies for five hepatocellular carcinoma (HCC) patients. They found that HCC treatment is associated with a high SCR in the lung and stomach (Kim et al., 2014). These studies and ours all attempted to predict the SCR for more efficient clinical decisions based on a mathematical model fitted from epidemiological data. The limitations of our study include the relatively small number of patients and model parameters, and the retrospective design. The performance of current risk models should be further improved to enable more precise estimation of the incidence of SCR; larger populations with long-term epidemiological studies are required. However, we overcame the time limitation and restricted clinical availability to provide a reference for the CK-LOT-SBRT SCR risk evaluation.

Review of the literature reporting SCR in patients who have received radiotherapy found that not all tissues have equivalent sensitivity to the carcinogenic effects of radiation. The highest incidences of SCR are lung cancers, and breast cancers in female patients, and there does appear to be a dose–response relationship (Followill et al., 1997; Kry et al., 2005; Parker et al., 2007). It is possible the SCR after CK-LOT-SBRT is significantly underreported. We are currently investigating whether SCR in the CK-LOT-SBRT results from higher doses of radiation to the sensitive tissues, to check whether the SCR risk meets the safe and effective dose criteria for patients. Through appropriate PTV coverage and OAR constraints, it may be feasible to decrease the dose to these sensitive tissues with CK-LOT-SBRT such that rates of secondary cancer could actually be lowered compared to conventional radiotherapies.

## Conclusion

CK-LOT-SBRT delivers precisely targeted radiation doses to lung cancers and achieves good PTV coverage and a conformal dose distribution, with low SCR for surrounding tissues. Younger patients had larger SCRs, whereas the dose–response relationship seemed be non-linear for the investigated OARs, especially in the case of the PTV. Despite the etiological association, the SCR after CK-LOT-SBRT for carcinoma and sarcoma is low, but not equal to zero.

Although the risk is small, with the ultimate treatment goal being to deliver safe and effective dosages to patients, physicians must be aware of the importance of identifying the SCR after CK-LOT-SBRT in the case of both carcinoma and sarcoma. It is possible that there is significant underreporting of SCR after CK-LOT-SBRT; it is unlikely that other identified cases have been published in the literature. Further research is required to understand and to show the lung SBRT SCR comparisons and differences across different modalities with motion management strategies.

## Data Availability Statement

All datasets generated for this study are included in the article/Supplementary Material.

## Ethics Statement

The studies involving human participants were reviewed and approved by The institutional review board of Chang Gung Memorial Hospital approved this study (IRB approval No. 201802377B0). Written informed consent for participation was not required for this study in accordance with the national legislation and the institutional requirements.

## Author Contributions

P-JC, I-HT, C-CH, C-SS, Y-WH, P-YY, H-FL, and C-HL contributed to the technical supports on data collection and analysis, analyzed the data and imaging processing, had provided valuable suggestions, and revised the manuscript. C-CH supported the characterization of the samples, had provided valuable suggestions, and revised the manuscript. P-JC and T-FL supervised the project, had given valuable advices on the proceeding of this work, designed the concept and the experiment method of the research, and wrote and revised the manuscript. All authors read and approved the final manuscript.

## Conflict of Interest

The authors declare that the research was conducted in the absence of any commercial or financial relationships that could be construed as a potential conflict of interest. Part of our results was presented in an abstract form at Phenma2019.
