# Supplementary material for: Radiation-Induced Secondary Cancer Risk Assessment in Patients With Lung Cancer After Stereotactic Body Radiotherapy Using the CyberKnife M6 System With Lung-Optimized Treatment
Source: Front Bioeng Biotechnol. 2020 May 7;8:306. doi: 10.3389/fbioe.2020.00306 (PMC7223476; doi:10.3389/fbioe.2020.00306)
Supplement: Supplementary file 1 [file Image_1.pdf]

Radiation-induced Secondary Cancer Risk Assessment in Patients with Lung Cancer after Stereotactic Body Radiotherapy using the CyberKnife M6 System with Lung-optimized Treatment

Pei-Ju Chao PhD[1,2], I-Hsing Tsai MS[1], Chun-Chieh Huang MD[2,*], Chih-Hsueh Lin PhD[1] , Chin-Shiuh Shieh PhD[1], Yang-Wei Hsieh MS[1,2], Pei-Ying Yang MS[1,2], Hsiao-Fei Lee PhD[1,2], Tsair-Fwu Lee PhD[1,2,3,*],

Supplementary Figures

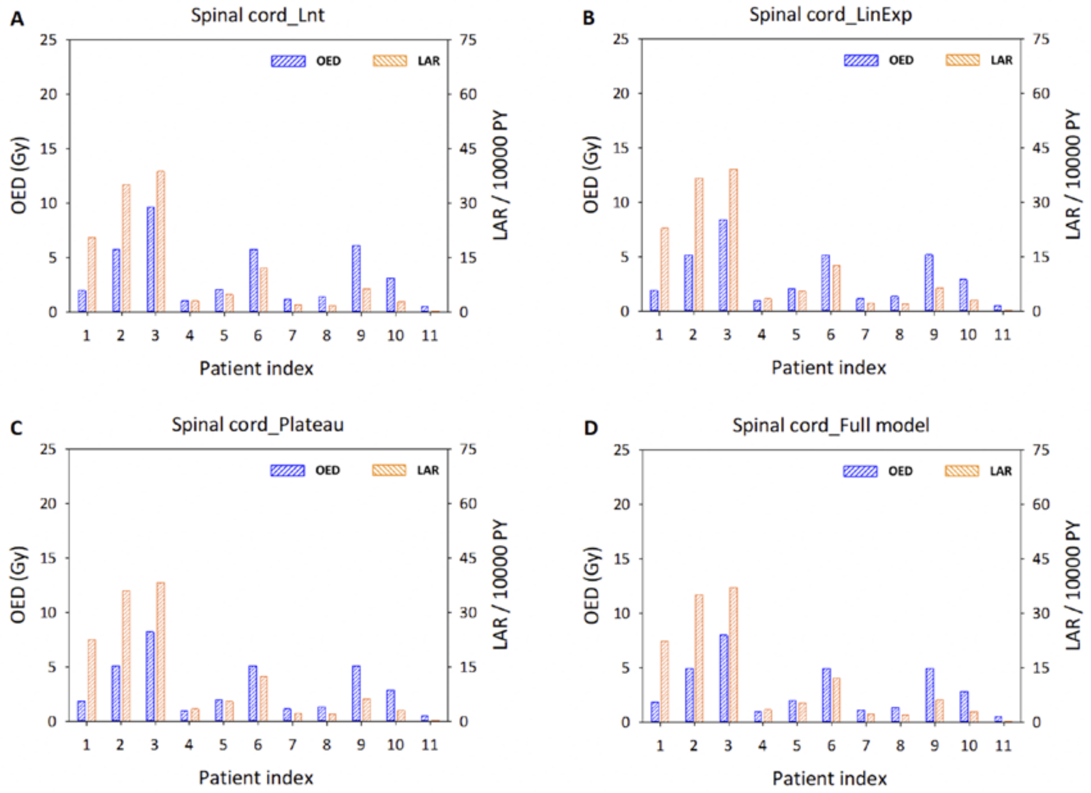

Supplementary Figure S1. OED/LAR of spinal cord for 11 patients using corresponding dose-response models **(A)** Lnt, **(B)** LinExp, **(C)** Plateau, **(D)** Full;

Notes: Patients were ordered by age from young to old; X axis: patient index; Y axis: OED (Left), LAR (Right); Unit is Gy (Left) and per 10,000 person-years (PY) (Right).

Abbreviations: Lnt, Linear-no-threshold dose response model; LinExp, Linear-exponential dose response model; Plateau, Plateau dose response model; Full, Schneider parameterization dose-response model; OED, Organ equivalent dose; LAR, Lifetime attributable risk; PTV, Planning target volume.

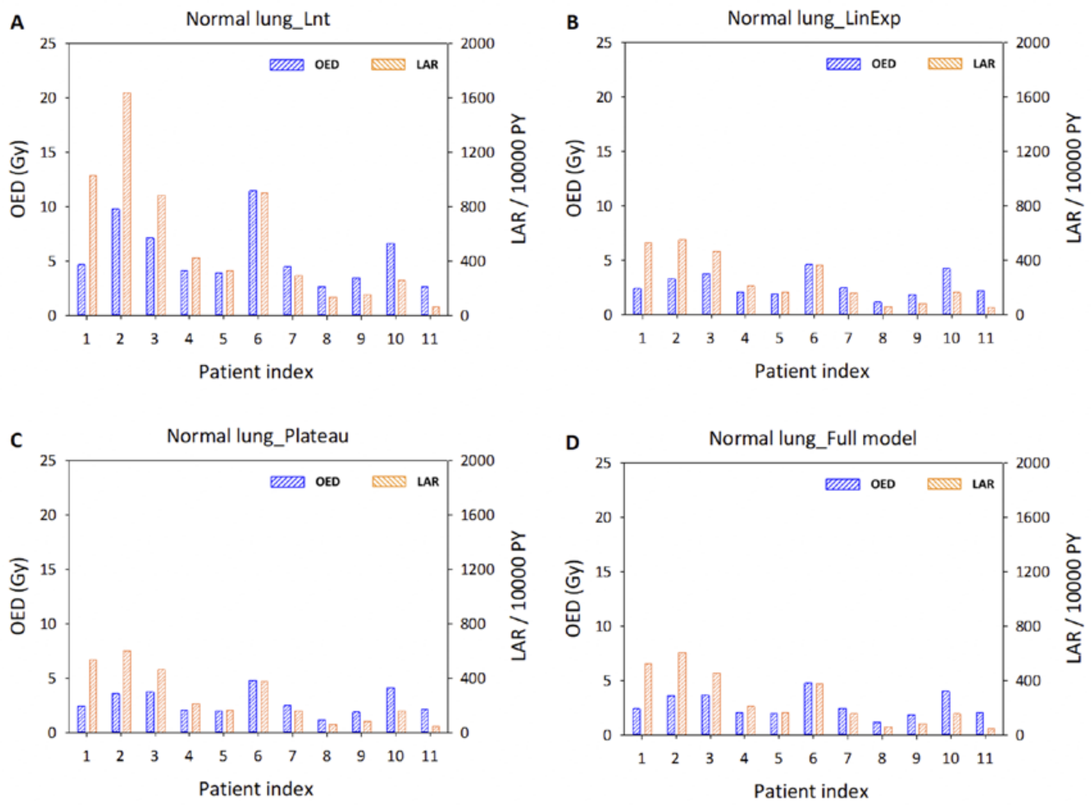

Supplementary Figure S2. OED/LAR of normal lung for 11 patients using corresponding dose-response models **(A)** Lnt, **(B)** LinExp, **(C)** Plateau, **(D)** Full;

Notes: Patients were ordered by age from young to old; X axis: patient index; Y axis: OED (Left), LAR (Right); Unit is Gy (Left) and per 10,000 person-years (PY) (Right).

Abbreviations: Lnt, Linear-no-threshold dose response model; LinExp, Linear-exponential dose response model; Plateau, Plateau dose response model; Full, Schneider parameterization dose-response model; OED, Organ equivalent dose; LAR, Lifetime attributable risk; PTV, Planning target volume.

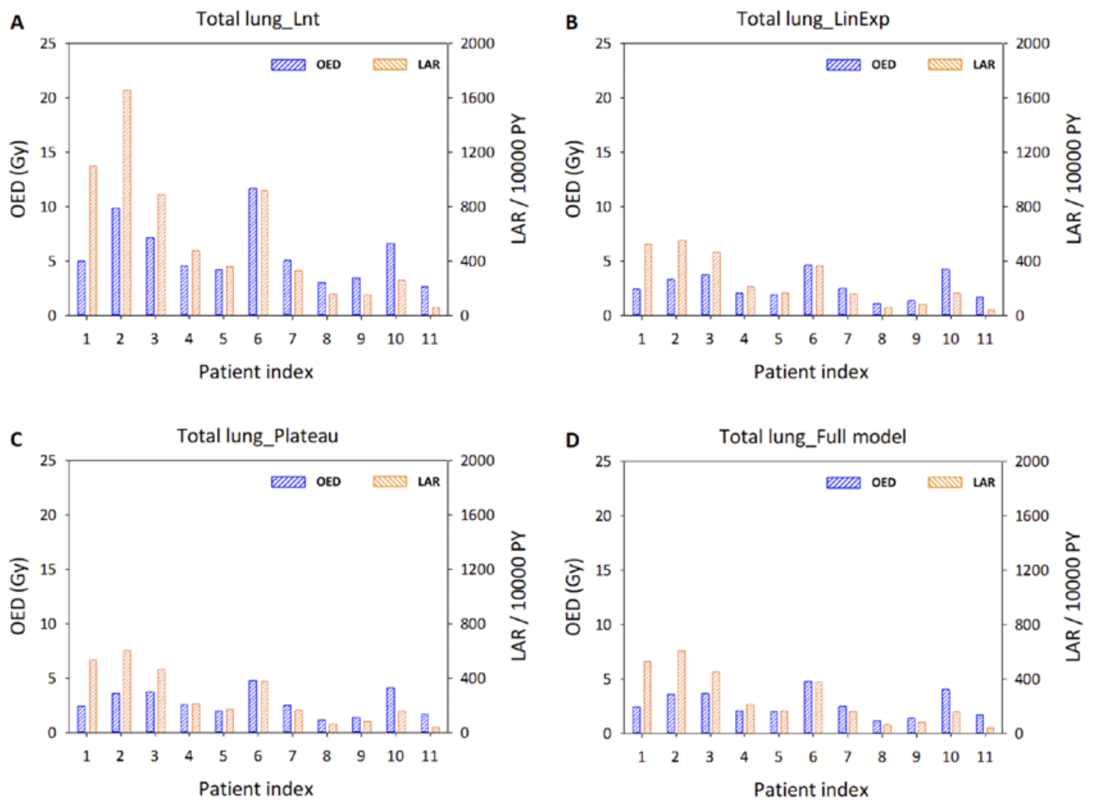

Supplementary Figure S3. OED/LAR of total lung for 11 patients using corresponding dose-response models **(A)** Lnt, **(B)** LinExp, **(C)** Plateau, **(D)** Full;

Notes: Patients were ordered by age from young to old; X axis: patient index; Y axis: OED (Left), LAR (Right); Unit is Gy (Left) and per 10,000 person-years (PY) (Right).

Abbreviations: Lnt, Linear-no-threshold dose response model; LinExp, Linear-exponential dose response model; Plateau, Plateau dose response model; Full, Schneider parameterization dose-response model; OED, Organ equivalent dose; LAR, Lifetime attributable risk; PTV, Planning target volume.

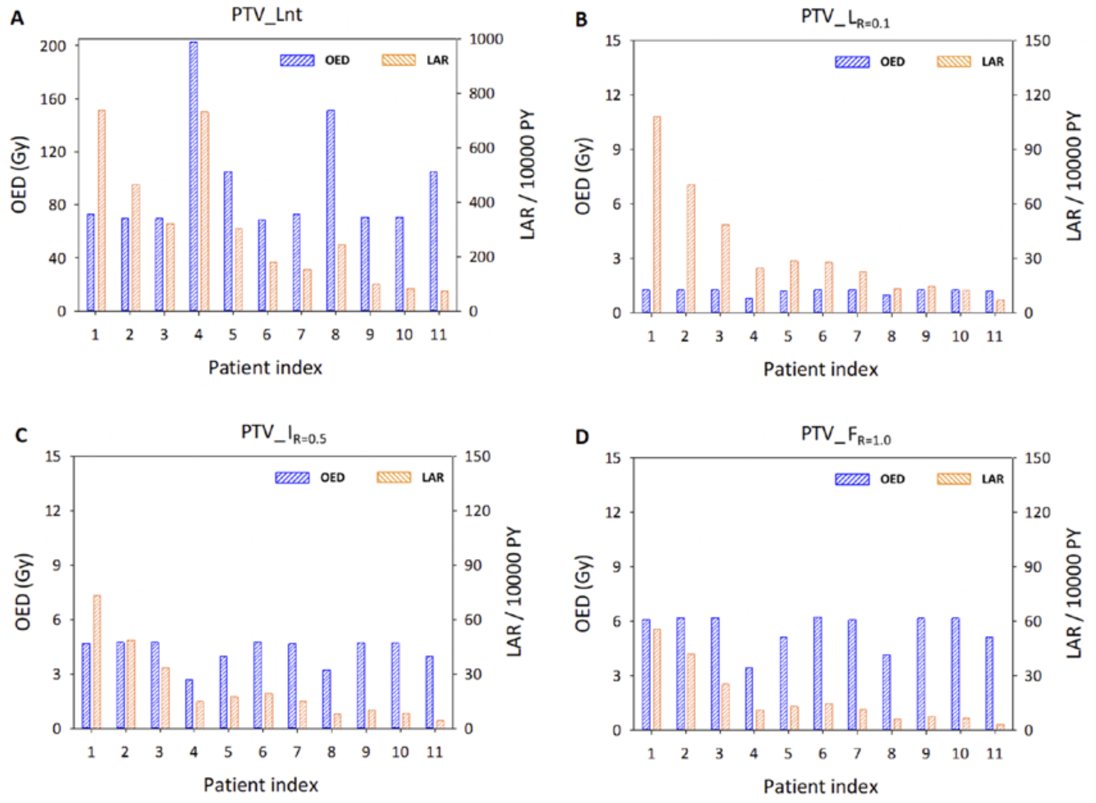

Supplementary Figure S4. OED/LAR of PTV for 11 patients using corresponding dose-response models **(A)** Lnt, **(B)** Low repopulation ($L_{R=0.1}$) R = 0.1, **(C)** Intermediate repopulation ($I_{R=0.5}$) R = 0.5, **(D)** Full tissue recovery models ($F_{R=1.0}$) R = 1.0;

Notes: Patients were ordered by age from young to old; X axis: patient index; Y axis: OED (Left), LAR (Right); Unit is Gy (Left) and per 10,000 person-years (PY) (Right).

Abbreviations: Lnt, Linear-no-threshold dose response model; Full, Schneider parameterization dose-response model; Low repopulation ($L_{R=0.1}$) R = 0.1, Intermediate repopulation ($I_{R=0.5}$) R = 0.5, Full tissue recovery models ($F_{R=1.0}$) R = 1.0; OED, Organ equivalent dose; LAR, Lifetime attributable risk; PTV, Planning target volume.
